# A switchable light-input, light-output system modelled and constructed in yeast

**DOI:** 10.1186/1754-1611-3-15

**Published:** 2009-09-17

**Authors:** Oxana Sorokina, Anita Kapus, Kata Terecskei, Laura E Dixon, Laszlo Kozma-Bognar, Ferenc Nagy, Andrew J Millar

**Affiliations:** 1 Institute of Molecular Plant Sciences, The University of Edinburgh, Kings Buildings, Mayfield Road, Edinburgh EH9 3JH, UK; 2 Institute of Plant Biology, Biological Research Center, Temesvari krt. 62, H-6726, Szeged, Hungary; 3 Centre for Systems Biology at Edinburgh, C.H. Waddington Building, Kings Buildings, Mayfield Road, Edinburgh EH9 3JD, UK

## Abstract

**Background:**

Advances in synthetic biology will require spatio-temporal regulation of biological processes in heterologous host cells. We develop a light-switchable, two-hybrid interaction in yeast, based upon the Arabidopsis proteins PHYTOCHROME A and FAR-RED ELONGATED HYPOCOTYL 1-LIKE. Light input to this regulatory module allows dynamic control of a light-emitting LUCIFERASE reporter gene, which we detect by real-time imaging of yeast colonies on solid media.

**Results:**

The reversible activation of the phytochrome by red light, and its inactivation by far-red light, is retained. We use this quantitative readout to construct a mathematical model that matches the system's behaviour and predicts the molecular targets for future manipulation.

**Conclusion:**

Our model, methods and materials together constitute a novel system for a eukaryotic host with the potential to convert a dynamic pattern of light input into a predictable gene expression response. This system could be applied for the regulation of genetic networks - both known and synthetic.

## Background

Gene expression systems with both spatial and temporal regulation are key components of engineered and synthetic biological networks. Engineered systems generally use a controlled external stimulus to signal to a specific promoter element, producing a rapid and dose-dependent response [1]. The external stimulus, used at the level of both the whole organism and cell culture, has often been a small, cell permeable molecule, which functions as an activator for the corresponding promoters [2-4]. Heat shock gene promoter systems can also be utilised for conditional gene expression using heat or irradiation as the stimulus [5].

The yeast artificial light switchable promoter system proposed by Shimizu-Sato *et al*. demonstrates many of the advantages of inducible systems, including low background expression, high inducibility, reversibility and dose-dependence [6]. It combines these desirable features with non-toxicity and a lack of pleiotropic and unanticipated effects which are inherent properties of chemically inducible systems. This system is based on the properties of the plant phytochrome B photoreceptor (PhyB), which reversibly changes its conformation in response to red (λ_max _= 660 nm) or far-red light (λ_max _= 730 nm). The far-red light absorbing conformer (PhyB Pfr) binds to the phytochrome interacting factor 3 (PIF3) protein, whereas interaction between the red light absorbing conformer (PhyB Pr) and PIF3 is much less efficient [7]. In the proposed system, PhyB and PIF3 are expressed as chimeric proteins, fused to the DNA-binding (GBD) or the transcriptional activator (GAD) domain of the GAL4 transcription factor, respectively, giving a typical two - hybrid interaction assay. The *cis *component of the system is the *lacZ *reporter gene controlled by a GAL4-responsive artificial promoter. In darkness, PhyB-GBD binds the promoter, but does not induce transcription. Red light illumination converts PhyB into the Pfr form, therefore facilitating PhyB-PIF3 interaction, which recruits PIF3-GAD to the GAL4-dependent promoter resulting in the activation of transcription. Subsequent far-red light illumination coverts PhyB Pfr to Pr and this is followed by the dissociation of the PhyB-GBD - PIF3-GAD complex and abrogation of transcription. The authors demonstrated the dose-dependent response of the system and the dynamics of photoreversible activation of the *lacZ *reporter gene, derived from quantitative liquid culture assays.

Recently, another genetically encoded signalling system based on PhyB - PIF3 interaction, with different chimeric proteins, has been successfully used for photoswitching of actin assembly through the Cdc42-WASP-Arp2/3 pathway in *E.coli *[8].

All phytochromes (PhyA-E) in the model plant *Arabidopsis thaliana *are capable of light-dependent conformational changes, but interacting proteins have only been investigated for the two most abundant phytochromes (PhyA and PhyB) [7,9,10]. FAR-RED ELONGATED HYPOCOTYL 1 (FHY1) and FHY1 LIKE (FHL) proteins control the nuclear import of PhyA via specific interactions with the Pfr conformer [11,12]. It follows that, besides the PhyB-PIF3 pair, other phytochrome-interacting protein combinations could be employed as the "light sensing" module of the expression system.

Functional phytochrome receptors consist of the apoprotein and the covalently linked chromophore called phytochromobilin. Since the chromophore is not synthesised in yeast, an analogous compound, phycocyanobilin (PCB), purified from cyanobacteria, is added to the media. PCB is taken up readily by yeast cells and is autoligated by phytochrome apoproteins resulting in photochemically functional phytochrome photoreceptors [13-15]. When expressed in yeast with PCB, PhyA behaves like other phytochrome receptors: the Pr ↔ Pfr conversion is controlled by red and far-red light [15-17].

The light switch described by Shimizu-Sato at al., translates light-dependent protein interactions into transcriptional regulation of a selected gene [6]. Beta-galactosidase is the most widely used reporter gene in yeast; however, the protein has a half-life of more than 20 hours in this system, and it can be detected *in vitro *only [18]. By comparison, the firefly luciferase has a 1.5 hour half-life in yeast, and luciferase activity (luminescence) can be monitored in real-time and *in vivo*, which makes this reporter a better tool for monitoring dynamic changes in transcription, as has been elegantly demonstrated recently through the monitoring of the cell-cycle and respiratory oscillations monitoring in agitated liquid yeast culture [19,20].

Our aim was to create and mathematically model an inducible gene expression system, based on the principles described above, but containing novel components that provide more stringent regulation and *in vivo *real-time detection of transcription in yeast colonies on solid media.

## Results

### Selection and testing of components for the light inducible expression system

**Figure 1:**
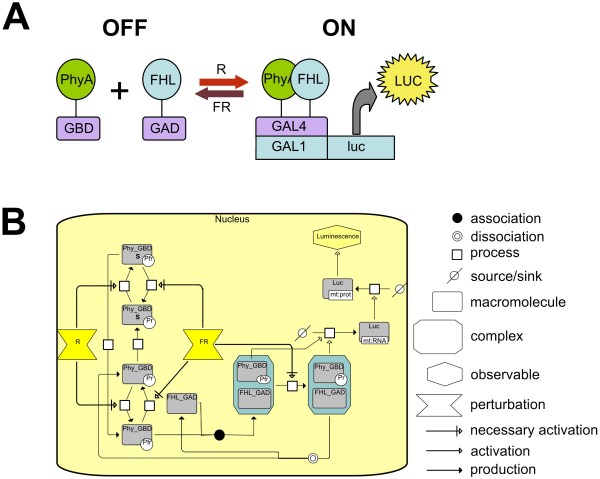
**Light-responsive gene promoter system**. Target gene expression is activated by red light, when photoactivated phytochrome A (Pfr_GBD) interacts with PFL_GAD and recruits it to the target promoter with Gal UAS sites for transcription activation. Expression of the gene could be switched off by far red application, when photoinactivated phytochrome (PR_GBD) dissociates from FHL_GAD. (A) Schematic representation. (B) SBGN representation [21]. Numbers correspond to following reactions: 1 Phytochrome Pr-Pfr photoconversion by red light (R). **2 **Phytochrome Pfr-Pr photoconversion by far red light (FR). **3 **Pfr-Pr dark reversion. **4 **Pfr dissociation from SAP (Sequestrated Areas of Phytochrome). **5 **Pr association to SAP. **6 **Phytochrome (Pfr_GBD) association with FHL_GAD. **7 **Pfr_FHL-Pr_FHL transition by FR **8 **Intermediate Pr_FHL complex dissociation. **9 **Transcription of luciferase gene. **10 **Translation of luciferase mRNA. **11 **Luciferin- luciferase enzymatic reaction

**Figure 2:**
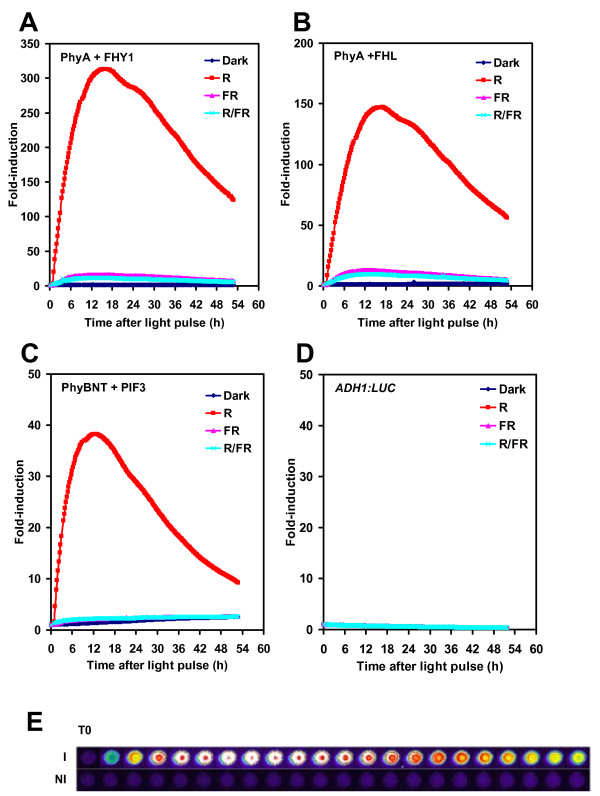
**Light switchable light output**. Yeast cells harboring the GAL1:LUC reporter and expressing PHYA-GBD/GAD-FHY1 (A), PHYA-GBD/GAD-FHL (B), or PHYBNT-GBD/GAD-PIF3 (C) fusion protein-pairs were grown in darkness to form patches (merged colonies) for two days at 30°C, treated with 2.5 mM luciferin and transferred to 22°C for 17.5 h. Separate yeast patches were irradiated with single red (R), or far-red (FR) light pulses, or with red pulses immediately followed by far-red pulses (R/FR), or were kept in darkness (Dark). Luminescence values normalised to the pre-pulse levels are shown; time 0 h is the start of the light treatment. The luciferin pretreatment is shown in Additional file 1. E: Selected luminescent images of yeast patches used to obtain data in panel A. I: red light-induced, NI: non-induced dark control, T0: last images before the light pulse. Consecutive luminescent images taken in every two hours are shown. Pictures are displayed in pseudo-colors: red-white or blue-black colors indicate high or low expression levels, respectively.

Detection of promoter induction via beta-galactosidase activity is a well characterised method in *S. cerevisiae *however it requires time-consuming sampling and *in vitro *analysis. In order to provide a real-time, *in vivo *detectable reporter in our system, the GAL4-responsive *GAL1 *promoter was fused to the firefly luciferase gene (*GAL1:LUC*) (Fig 1). Figure 1B shows the resulting gene circuit in the community standard Systems Biology Graphic Notation (SBGN) [21]. As a constitutive control, the *ADH1:LUC *(ALCOCHOL DEHYDROGENASE I) construct was prepared and stably integrated into the genome. Yeast colonies prepared as described in Materials and Methods reached a steady state of luminescence 16-18 hr after luciferin was applied (Additional file 1). As expected, *ADH1:LUC *produced much higher light emission than *GAL1:LUC *independent of the GBD/GAD fusion proteins expressed (Additional files 1 and 2). Separate set of patches were irradiated with red light (R) or far-red light (FR), or R immediately followed by FR (R/FR), or were kept in darkness. R light induced a rapid increase of luminescence in the case of yeast patches expressing *GAL1:LUC*, but not in the *ADH1:LUC*-expressing patches or in patches expressing *GAL1:LUC *without GBD/GAD fusion proteins (Fig 2 and Additional files 1 and 2). Luminescence reached a maximum 14-16 hr after the R light, followed by a slow decrease. In contrast, FR light alone induced very low levels of luciferase activity, which was essentially the same, when R light treatments were followed by FR light immediately (Fig 2). Since the relative (fold) induction was the highest in yeast cells having the *GAL1:LUC *reporter and expressing PHYA-GBD and FHY1-GAD (Fig 2A), this set of interacting proteins were used in further experiments. These results demonstrate that (i) appropriate LUC markers can be used to report phytochrome photoconversion and light-induced protein-protein interactions in our system; (ii) LUC enzyme activity is unaffected by light in yeast and (iii) yeast patches grown on solid media and treated with luciferin represent stable and reliable experimental material for luminescence imaging.

### Luciferase as a reporter for gene expression in yeast

In order to calculate changes in the rate of transcription from real-time luminescence data, it was necessary to determine the relationship between transcription and enzyme activity.

**Figure 3:**
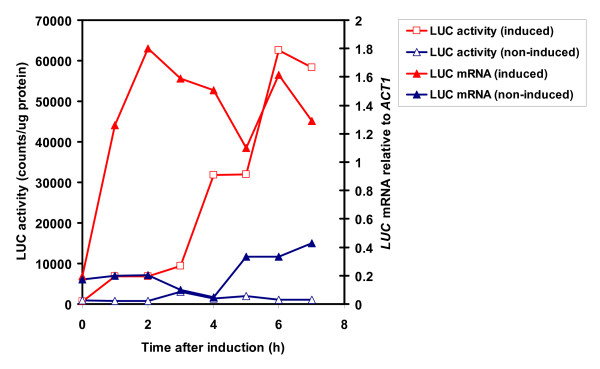
**Kinetics of induction of luciferase mRNA and luciferase activity from the *CUP1:LUC *reporter gene**. Yeast cells harboring the *CUP1:LUC *construct were grown in liquid rich media overnight at 30°C. *CUP1:LUC *expression was induced by 1.5 mM copper-sulphate (final concentration) at time = 0 and aliquots were harvested hourly. The samples were used to prepare crude protein extracts and to isolate total RNA. Luciferase activity was measured by in vitro assays and luciferase mRNA was determined by qRT-PCR reactions.

The copper inducible *CUP1 *promoter was fused to luciferase gene, expression was induced and *CUP1: LUC *RNA and luciferase activity were tested over 7 hours time course. Figure 3 shows a 3-4 h delay in the induction of LUC activity relative to *LUC *mRNA expression. These data contributed to determine the kinetic parameters (mRNA half-life, translation rate) for the Luc reporter model.

### An unidentified compound functions as a chromophore Text for this sub-section

**Figure 4:**
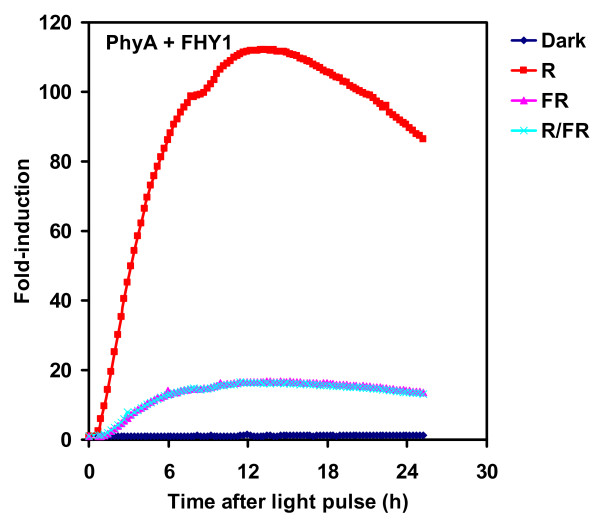
**Switching without PCB**. Yeast cells harboring the *GAL1:LUC *reporter and expressing PHYA-GBD and GAD-FHY1 fusion proteins were grown in darkness at 30°C for two days on media without PCB. Luciferin and light treatments and imaging were performed as in Fig. 2A.

Phytochromes are chromoproteins consisting of the apoprotein and a covalently linked, linear tetrapyrrole chromophore, phytochromobilin (PΦB) [22]. In the absence of chromophore, phytochromes cannot absorb light, do not show light dependent conformation changes and, therefore, do not function as photoreceptors. Phytochrome apoproteins are synthesised in the Pr form in plants and after autoligation of PΦB are capable of light absorption and photoconversion into the Pfr conformer. Cyanobacteria (Synechococcus and Synechocystis sp.) harbour phytochrome-like photoreceptors, which use a chromophore (PCB) with similar structure to that of PΦB [23]. Plant phytochromes binding PCB are fully functional photoreceptors and, because of the relative ease of PCB purification it is generally used as an exogenously added chromophore [24]. However, it was unclear whether yeast cultures contained chromophore-like compounds that could serve as the chromophore for plant phytochromes. To test this, yeast cells with the *GAL1: LUC *reporter and expressing PHYA-GBD and FHY1-GAD were grown on media lacking PCB and the same light treatments were administered as in Fig 2A. To our surprise, significant R induction was detected in the absence of PCB (Fig 4). The fold-induction was reduced to 30% compared to the results with added PCB (Fig 4 vs. Fig 2A). Moreover, Figure 4 shows that FR light alone, or R followed by FR light also gave qualitatively very similar results to the photoreceptor with PCB. The basal expression level of *GAL1:LUC *was not affected significantly by the presence or absence of PCB (Additional file 2). These results demonstrate that an unidentified compound naturally present in yeast, can serve as a chromophore for phytochromes expressed in this heterologous system.

### Model assumptions and structure

To use our regulatory system for synthetic biology we developed an ordinary differential equation (ODE) model of its function based on kinetic data from the literature and experimentally determined parameter values (Fig 1A, B and Additional file 3). The model describes all the known phytochrome properties (e.g. photoconversion, dark reversion, sequestration, etc), using yeast phytochrome data to provide a realistic description of the light-switch function (for the detailed model description and structure, see Methods).

In summary, the model assumptions are:

1) Overall concentrations of Phys and PIF3/FHY1/FHL are constant,

2) Before the light impulse all the phytochromes are in the inactive (Pr) form, and sequestered in a slow acting pool. This might be related to their inclusion in SAPs(Sequestrated Areas of Phytochromes)-like structures similar to those observed by microscopy in cytosol [15] (see Methods for more details).

3) The dark reversion rate is the same for the free Phy and the Phy-FHY1/FHL complex.

4) Photoconversion or dark reversion of the Pfr_FHY1/FHL complex creates an intermediate step (Pr_FHY1/FHL) with distinct kinetics, prior to dissociation of the complex.

5) The luciferase - luciferin subsystem is approximated as a steady state before light treatments.

6) The initial sharp decrease in luminescence following the application of luciferin is due to the diffusion of luciferin from the point of application on the yeast patch into the agar medium.

This enables the model to provide a good fit to conceptually similar systems with different interaction partners. For example, the adapted model fits Shimizu-Sato's data with good accuracy using parameter values derived from the literature [6,7,16,25]. This model is simpler, for the main part because the slow LacZ degradation obscures the long-term kinetics (see the model equations in Methods and simulation results in Additional file 4).

**Figure 5:**
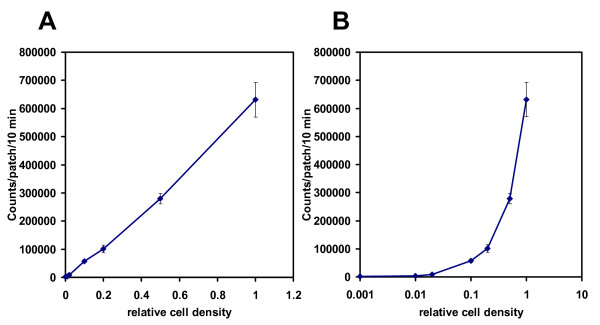
**Effect of cell density on LUC luminescence intensity**. Yeast cells harboring the *ADH1:LUC *reporter were grown in liquid culture at 30°C for 16 h. Dilution series were prepared from the overnight culture and 20 μl of each dilution were pipetted on solid media and allowed to dry for 15 min at 22°C. The patches were treated with 2.5 mM luciferin and imaged subsequently. Average luminescence values of three replicates for each dilution were plotted against the relative cell density. Graphs with linear (A) or semi-logarithmic (B) scale are shown. Error bars represent standard deviation. The culture with 0.01 relative cell density had OD260 = 0.232 and the corresponding patches contained 8.2·10^4 ^cells. Yeast cells were counted by a Burker counting chamber.

**Figure 6:**
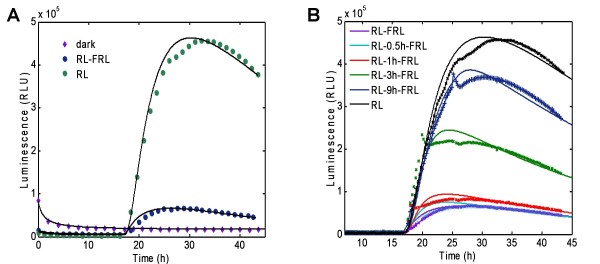
**Time course of luciferase luminescence intensity in different light conditions**. Experiment results (markers) and simulation (solid lines) are presented at the same scale. (A) Yeast cells containing PhyA_GBD and FHL_GAD were incubated in the dark. Luciferin was added at time zero with application of: no pulse (dark),10 min red pulse (RL), or 10 min red pulse followed immediately by 10 min far red pulse (FRL) at 18 h. (B) On-Off experiment with different time intervals between red and far red pulses. Yeast cells containing both PHYA-GBD andd FHL-GAD were preincubated with PCB in the dark. Luciferin was added at time zero. At time 18 h all the cultures were given the 10 min red pulse (RL) or 10 min red pulse with subsequent 10 min far- red pulse at 0 (RL-FRL), 0.5 (RL-0.5 h-FRL), 1 (RL-1 h-FRL), 3 (RL-3 h-FRL) and 9 h (RL-9 h-FRL) after red pulse. A representative dataset of 5 replicates is shown.

To account for the initial difference in cell density for each yeast patch that affects luminescence intensity (Fig 5) and for the non-uniformity of the solid media, the initial conditions were set for each patch (each experiment) individually, so that each experimental curve is considered for two regimes, "diffusion" and "phytochrome". The former starts from the application of luciferin with initially decreasing luminescence, which approaches an approximate steady state after 17-18 hours. The latter begins from the light treatment and continues to the end (Fig 6A). The first regime fits separately to the diffusion part of the model and provides the initial luciferin level at the time of light application. Such decomposition of the initial conditions is introduced to describe the temporally changing substrate availability that emerges from the solid culture conditions. Modelling the solid media allows a much wider variety of experimental applications in conditions that are most common in yeast and synthetic biology. The diffusion coefficient in our experiments corresponds to diffusion rate of 3 to 10 mm/h, which is in good agreement with literature data for agar gel [26].

Parameter values for model equations were obtained from fitting the model to all the timeseries data from luciferase imaging (Fig 6A, B), within the parameres ranges derived from the literature (Table 1). The parameter data for luciferase protein degradation rate was supported by additional experiments using cycloheximide treatment of yeast cultures constitutively expressing the luciferase gene (data not shown). The degradation rate constant is estimated to be 0.2-0.8, which corresponds to a 0.8-3 hour half-life. This is similar to the value measured in yeast and mammalian cell cultures and plants [27-29].

**Table 1:** Final parameter set for PhyA-FHL model

**Parameter description**	**Parameter name**	**Parameter value**	**Dimensions**	**Literature data**
Total PhyA concentration	Pool_PhyA	4.93E+01	nM	

Total FHL concentration	Pool_FHL	1.00E+02	nM	

Rate of assocoation for PhyA and FHL	K1	1.50E+00	1/nM*h	

Rate of basic (background) transcription for GAL4 promoter	k_base	4.50E-01	nM/h	

Rate transition to free pool	K2	1.00E-02	1/h	

Rate of sequastration to SAPs	K3	9.90E+00	1/h	

Rate of Pr_FHL complex dissociation	K_dis	3.00E-02	1/nM*h	

Rate of dark reversion	K_rev	2.00E-01	1/h	0.027-1.2 [14,35]

Rate of photoactivation by R light	Ka_R	4.96E-03	m2/um	4.96E-03 [30]

Rate of photoactivation by FR light	Ka_FR	3.55E-05	m2/um	3.55E-05 [30]

Rate of photoinactivation by R light	Ki_R	7.44E-04	m2/um	7.44E-04 [30]

Rate of photoinactivation by FR light	Ki_FR	1.70E-03	m2/um	1.70E-03 [30]

Rate of luciferase RNA degradatin	m_luc	3 (12 min)	1/h	

Rate of luciferase translation	p_luc	1.00E+01	1/h*nM	

Rate of luciferase protein degradation	m_LUC	0.8 (48 min)	1/h	0.23-1.3 [28,29]

Rate of the luciferin-dependent luciferase degradation	m_LUC1	0.00E+00	1/h	

Luciferase activity constant	k_kat	4.99E+02	nM/h	144 [28]

Michaelis constant for luciferin	Km	1.90E+00	mM	0.1-2 [28]

Hill coefficient for transcription	a_luc	2.00E+00		

Maximum transcription rate	n_luc	2.94E+01	nM/h	

Light conversin factor	RLU	1.00E+01		

Michaelis constant for transcription	g_luc	1.04E+00	nM	

### Model predictions

**Figure 7:**
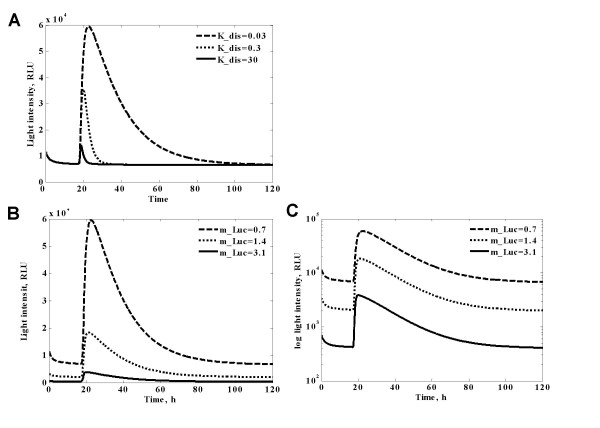
**Investigation of the crucial parameters by simulation of the model**. **A**. Simulation of different stability of Pr_FHL intermediate state. Dashed line corresponds to initial value of dissociation constant K_dis, derived from fitting to the experimental curves. Dotted line corresponds to 10 time's faster Pr_FHL complex dissociation. Solid line corresponds to 100-times faster complex dissociation. **B**. Simulation of different stability of Luc reporter. Dashed line corresponds to initial value of degradation rate constant m_luc, derived from fitting to experimental data. Dotted line corresponds to twice faster degradation rate of luciferase. Solid line corresponds to more than 4-times faster degradation rate. **C**. Same as B, but in semilog scale. Reporter instability affects the amplitude of the signal but not the overall timing of events

The refined model both captures qualitative dynamics and enables a quantitative description of the light switching behaviour. Moreover, it enables us to deduce which parameters would be critical for particular behaviours of the system. By varying these parameters we showed that predicted intermediate state during the photoconversion of Pfr_FHL is crucial to match the slow switching off of the observed LUC expression (Fig 7A). The biochemical nature of this state as well as its experimental measurement is the subject of further experiments. Also, according to the model simulation (Fig 7B, C), shortening of reporter protein half-life does not affect the longevity of reversal of the transcription activation but significantly reduces the intensity of the luminescence.

**Figure 8:**
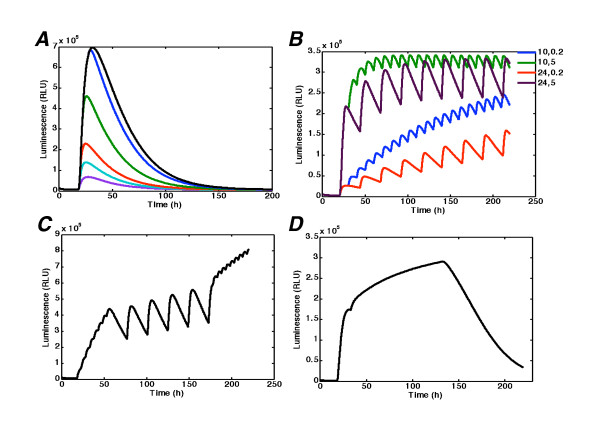
**Model simulation and predictions**. (A) Single peaks. Long term On-Off model simulation with far red light application in different time intervals after red pulse. Luciferin was added at time zero. At time 18 h all the cultures were given the 10 min red pulse or the 10 min red pulse with a subsequent 10 min far-red pulse at 0, 0.5, 1, 3 and 9 h after the red pulse. (B) Multiple peaks. Model simulation with different time intervals between red and far red pulses. The green curve corresponds to 10 h between RL pulses and 5 h between RL and FRL pulses, the black curve to 24 h between RL pulses and 5 h between RL and FRL pulses, the blue curve to 10 h between RL pulses and 2 min between RL and FRK pulses, and the red curve to 24 h between RL pulses and 2 min between RL and FRL pulses. (C) Possible profile of transcription activation with RL pulse at time 18 h and subsequent RL-FRL pulses with 2 h between RL and 2 min between RL-FRL until time 133 h-"square" shape. (D) Possible profile of transcription activation with RL and FRL pulses of different frequencies of RL (RL-FRL is 2 min everywhere):5 h-24 h-5 h-"sigma" shape

The light switch model also gives several predictions about the long-term system behaviour (Fig 8A). In particular, we predicted based on experimental data for 50 h, that in the experimental conditions considered the complete removal of the transcriptional activation effect should take a relatively long time (100 hours), and this has been confirmed by experiments (data not shown). Furthermore, with the given dynamics, we can manipulate subsequent applications of R and FR, to achieve a wide range of desirable profiles of transcription activation (Fig 8B, c, and 8D). Fig 8B illustrates the different types of behaviour of light input, which depend on the interval between R and FR treatments. It is clear from simulations that a small interval (2 min) between R and FR causes the increase of transcription rate and, accordingly, the increase of the luminescence intensity with time. Meanwhile, a longer interval (5 hours) produces the stable base line of input oscillations. On the basis of these simulations one can create a specific protocol of light input, combining the given modes as required, and thus obtaining the "square" shape (Fig 8C) with two modes of light regime, and "sigmoid" shape (Fig 8D) with three modes. Thus, the overall system could be used as a tool for the design of experiments with flexible perturbations of the system, for example, by changing the time intervals between.

## Discussion

We developed a photo-regulatory genetic switch for yeast cells that combines several desirable properties. In addition to the widely recognised interacting pair PhyB-PIF3 we have tested other possible protein combinations. We found that the PhyA-FHY1 (and PhyA-FHL) pair provides higher induction level with lower background than that of the PhyB-PIF3 pair in our experimental conditions.

Previous experiments were carried out using agitated liquid yeast cultures at 30°C [6]. We used yeast colonies grown on solid media, for the reason that this setup facilitates light treatments, continuous monitoring of luminescence and potentially allows spatial patterning of light input and biological response. Our experimental system represents a reliable, reproducible and simple set up for investigation of dynamic transcription.

Phytochrome photoperception in yeast has previously been reported with addition of exogenous chromophore (PCB) [6,11]. Our system also showed light responsiveness without exogenous chromophore, albeit at a lower level (Fig 4). We propose that phytochromes can employ an unidentified compound from yeast as a chromophore, but the light absorbing efficiency of the constituted receptor is less than that of the holoprotein binding PCB. As a result, R treatment induces a reduced amount of phytochrome Pfr, which results in less efficient induction of transcription. The heterologous chromophore may be specific for some yeast strains, or its weak activating effect could have been difficult to detect using previously-employed reporter genes.

We developed a mathematical model that describes the system and fits the experimental data with great accuracy. The model incorporated experimental variability arising from the cultures on solid media, *via *substrate diffusion that corresponds to observations and sets the initial substrate concentration. To our knowledge, this is the first attempt to model the specificity of substrate distribution in solid cell culture. The model results demonstrate good overall accuracy (see Fig 6A, B); nevertheless, the success of the fit varies between experiments. For example, in the On-Off experiments (Fig 6B), model fits better to the longer intervals (at least 1 hour between R and FR treatments), compared with the shorter treatment intervals, when FR is given immediately or 30 min after R. It can be seen from the time series that there is only a small quantitative difference between immediate and 30-min-delayed FR, while after a 1 hour delay the shape of the response resembles that of a single R treatment (Fig 6B), differing only in amplitude. This qualitative shift in the system behaviour requires further analysis, which may shed light on mechanism of transcription activation by R light and inactivation by FR light in the system.

We found that the kinetics of induction were slower in our conditions compared to previously reported experiments [6]. For our tests, yeast patches were grown at 30°C for two days, and then, due to technical issues, the plates were moved to 22°C in the imaging chamber, so the effect of light treatments was investigated at 22°C. We found evidence that the system responds more quickly at 30°C (data not shown), but a complete explanation requires further investigations.

Our system does not display an instantaneous shutting off of target gene expression. It takes a substantial period of time to completely remove the Luc signal after FR treatment. Modelling suggests that this is not simply due to stability of the Luc reporter (see Fig 7B, C), but rather reflects persistent PhyA activity. It should be noted that far-red exposure does not convert all the active PhyA into Pr form, but by itself produces about 3% of Pfr form [30]. Additionally, we propose a residual physical interaction between the Pr form of PhyA and FHY1/FHL as a possible explanation for the slow kinetics. This was supported with model simulations that correspond to the experimental kinetics. However, the properties of the intermediate state remain to be determined.

## Conclusion

The current work initially aimed to create a system to provide well-defined, light-induced perturbations in transcription to a genetic oscillatory circuit, to effect entrainment of the oscillations to a rhythmic light regime. The light switchable system presented here meets the requirements for an entrainment tool; moreover the mathematical model will facilitate the design of any desired entrainment mode. Hence, using the light switch with the corresponding model provides a powerful tool for regular perturbing any gene system of interest with a predictable amplitude and period. Moreover, with spatially-patterned light inputs, such as images, the system would allow spatio-temporal regulation, which could facilitate a greater understanding of biological processes in which inter-cellular communication is involved.

## Methods

### Constructs, yeast strains and growth conditions

Plasmids expressing PHYA-GBD, PHYBNT-GBD, GAD-PIF3, GAD-FHY1 and GAD-FHL fusion proteins have been described [6,11,12]. PHYBNT corresponds to an N-terminal fragment of PHYB containing residues 1-621. *GAL1*, *CUP1 *and *ADH1 *promoters containing full 5' un-translated regions and the 3' un-translated region (terminator) of the *GAL2 *and *ADH1 *gene were amplified from S. cerevisiae PJ69-4A genomic DNA using the following primers:

GAL1 Fwd: 5'-AAAGTCGACATTACCACCATATACATATCC-3'

GAL1 Rev: 5'-TTGAATTCTCTCCTTGACGTTAAAGTAT-3'

CUP1 Fwd: 5'-CGGGTCGACGCCTTGTTACTAGTTAGAAAAAGACATT-3'

CUP1 Rev: 5'-CGCCGAATTCTTTATGTGATGATTGATTGATTGATT-3'

ADH1 Fwd: 5'-CTGGGATCCGCTGCAGGTCGAGATCCGGGATC-3'

ADH1 Rev: 5'-CGCGAATTCTGGAGTTGATTGTATGCTTGGTATAGCTTGA-3'

GAL2t Fwd: 5'-ATACTGCAGTGCGTTTGAAGTGAGACGC-3'

GAL2t Rev: 5'-ATACCCGGGTGGAAGAAAGTCCAGGCAAG-3'

ADH1t Fwd: 5'-CGCCTGCAGAGCTTTGGACTTCTTCGCCAGAGGTTT-3'

ADH1t Rev: 5'-TATCCCGGGGGCCGGTAGAGGTGTGGTCAATAAGAGC-3'

The firefly luciferase gene was amplified from plasmid pGL3 (Promega) using the following primers:

LUC Fwd: 5'-ATGAATTCATGGAAGACGCCAAAAACATA-3'

LUC Rev: 5'-ATACTGCAGTTACACGGCGATCTTTCC-3'

The promoter:luciferase-terminator constructs were assembled in pBluescript SK plasmid using the restriction sites designed for the PCR primers (sites are underlined in the sequences of the primers above). All plasmids were transformed into *E.coli *by the SEM method and cultured under standard conditions [31]. The *GAL2 *or the *ADH1 *terminator was used for the *GAL1*, *CUP1:LUC *or the *ADH1:LUC *construct, respectively. The constructs were verified by sequencing and re-cloned in the integrating plasmid pδ-UB [32]. The final clones were linearized with XhoI and transformed in yeast strain PJ69-4A by standard LiAC/carrier DNA/PEG protocol. Transformants were plated on synthetic dropout media (SD) without uracil SD(-U). Selected strains carrying the *GAL1:LUC *construct were co-transformed with plasmids pD153 or pGADT7 (Clontech) expressing GBD- or GAD-fusion proteins, respectively [6]. Transformants were selected and maintained on SD(-LW) plates. Preparation of media and transformation of yeast cells was done according to the Clontech Yeast Protocols Handbook (Clontech).

### In vivo luminescence imaging, light treatments

2 ml of selective SD media was inoculated with yeast cells and incubated for 16 hr at 30°C with agitation. 20 μl drops of the cultures were transferred to SD agar plates containing 10 μM PCB, irradiated with far-red light at 70 μmolm^-2^s^-1 ^fluence rate for 10 min and incubated for 48 hr at 30°C in darkness. All further manipulations were conducted under green safety light. Yeast cells formed merged colonies (or patches) with 5-8 mm diameter. 20 μl of 2.5 mM luciferin solution was pipetted at the center of each patch and the plates were transferred in the imaging chamber at 22°C. Images were taken every 15 minutes using a liquid nitrogen-cooled CCD camera (Visitron Systems GmbH, Munich, Germany). Luminescence was quantified using the Metamorph software (Molecular Devices, Downingtown, PA). Unless stated otherwise, light treatments were administered 17-18 hr after the application of luciferin. The duration of each light treatment was 10 min and fluence rate of light (independent of wavelength) was 70 μmolm^-2^s^-1^. Red and far-red light was provided by Snap-Lite LED modules (Quantum Devices, Barneveld, WI).

### Induction of CUP1:LUC expression, qRT-PCR and in vitro luciferase assays

Yeast cells carrying the *CUP1:LUC *construct were inoculated in 10 ml of SD(-U) media and were grown for 16 hr at 30°C with agitation. The starter cultures were diluted to a final volume of 100 ml with fresh SD(-U) media. *CUP1:LUC *expression was induced by adding CuSO_4 _solution to a final concentration of 1.5 mM. Samples were harvested hourly from induced and non-induced cultures. 2 ml or 100 μl of the cultures were pelleted and frozen for RNA quantification or for luciferase assays, respectively. Total RNA was isolated by using the RNeasy Plant Mini Kit (QIAGEN) according to the manufacturer's instructions. cDNA synthesis and qRT-PCR was performed as described [33]. Primers for qRT-PCR were:

LUC Fwd: 5'-GGAGCACGGAAAGACGATGACGG-3'

LUC Rev: 5'-ACAAACACAACTCCTCCGCGCA-3'

ACT1 Fwd: 5'-CACCAACTGGGACGATATGGA-3'

ACT1 Rev: 5'-GGCAACTCTCAATTCGTTGTAGAA-3'

Luciferase-specific signals were normalised to ACTIN 1 (ACT1) levels for each sample. For *in vitro *luciferase activity measurements, frozen cell pellets were re-suspended in 100 μl of Cell Culture Lysis Buffer (Promega), and vigorously vortexed. After incubation on ice for 5 min, cell debris was pelleted by centrifugation and the supernatant was used as crude protein extract. 20 μl of protein extracts was mixed with 30 μl of the Steady-Glo Luciferase Assay Reagent (Promega) in the wells of a microtiter plate and luminescence was measured in the TopCount NXT luminometer (Perkin-Elmer) for an hour after the addition of the reagent. Counts during monitoring were averaged and normalized to total protein content of the extracts. Protein concentrations were determined by the Bradford assay [34].

### Modelling

#### 1. Model description and structure

Our principal model system (Fig 1A, B) includes two chimeric proteins: phytochrome fused to the GAL4 DNA-binding domain (Phy_GBD), in the active (Pfr) and inactive (Pr) forms, and binding protein PIF3 (or FHL/FHY1) fused to the GAL4 activation domain (FHL_GAD). According to existing experimental data, the recombinant phytochromes are quite stable in yeast. Although the light lability of plant PhyA Pfr is well-described, no detectable difference was observed between the stability of the Pfr and Pr forms of oat PhyA over an 80 hour time period in yeast [24]; moreover, no significant decay in the total PhyA and PhyB amounts over 120 hours was reported [35,15]. This provides the basis for assuming that our model proteins are present constitutively, so neither production nor degradation occurs in the model system. Two pools, Pool_Phy and Pool_PIF3, fulfil the mass conservation laws for Phy and PIF3.

In plants, the Pr forms of phytochromes are localized in the cytoplasm in the dark and are translocated to the nucleus in their Pfr form after light absorption [36]. In the yeast system, however, all fusion proteins are constitutively nuclear-localized due to the natural Nuclear Localisation Sequence (NLS) present in the GBD tag or the presence of the SV40 NLS motif fused to the GAD fusion partner. Therefore, in this system the only light-dependent event is the interaction of phytochromes with their corresponding protein partners. Taken together, these details give us reason to locate the interacting proteins and the processes of association and dissociation in the nucleus.

Not instantaneous kinetics of induction (Fig 2) prompted us to suggest the existence of two phytochrome pools: slow and fast. It has been reported that the sequestration of recombinant PhyA into the cytosolic SAPs (sequestered areas of phytochrome) in yeast has no dependence on light [15]. We, therefore, propose the presence of sequestered and free Phy pools (less and more easy to access, respectively) in the nucleus with a reversible interchange occurring between them. We assume that only the free pool is available for binding to its interaction partner, and, thus, the transition between slow (sequestrated) and fast (free) pool is responsible for the shape of initial light response.

It is well known that the phytochrome photoconversion cross-section (σ) for Pr and Pfr forms depends on the wavelength of light. Red (approximately 660 nm) and far red (approximately 730 nm) light are the most effective for Pr → Pfr and Pfr → Pr photoconversions, respectively. Nevertheless, it is evident from the cross-section data that the absorption spectra of the Pr and Pfr forms of Phy significantly overlap [30]. This means that monochromatic light of biologically relevant wavelength (i.e. red) does not convert all the Phy to the Pr or Pfr form, but rather determines the specific distribution ratio of the forms in the total Phy pool. We thus have to account for the activation and inactivation of phytochrome by both red and far-red light, so that:



Exact values for Ki and Ka for the different wavelengths were adopted from [30]. In the model Pr ↔ Pfr transitions are applied for both associated and free form of the phytochromes.

Dark reversion has been reported for PhyA and PhyB in yeast cultures [15,35,13]. According to these data, only a fraction of the total Pfr pool is subject to dark reversion (20-40% of the total amount) with a half-life of 20-40 min. For simplicity in the current model we assume a single Pfr pool that is dark reversible and has a longer half-life than the range suggested by Hennig et al [13]; however, the model is still in good agreement with the overall kinetics described in the literature [35,15].

We assume that dark reversion of the complex Pfr_PIF3 (Pfr_FHY1, Pfr_FHL occurs with the same rate. Therefore, both the photoconversion and dark reversion processes contribute to dissociation of the transcriptional activation complex.

Finally, for the PhyA_FHY1/FHL complexes, we assume the existence of an additional state, Pr_FHY1/FHL, that has the ability to activate transcription to some extent, as it has been previously demonstrated by [11]. Although the reference above corresponds to PhyA, in our experimental conditions PhyB demonstrated the same kinetics (Fig 2C), so we assume the intermediate state for PhyB-PIf3 as well. According to our hypothesis, this complex is produced as an intermediate product of photoconversion of the Pfr_FHY1/FHL complex after FR exposure. Thus, we propose that Pr proteins that have previously been Pfr can interact with FHY1/FHL and activate transcription.

Mass Action kinetics were used to describe complex formation and dissociation, translocation, translation, and degradation. Transcription was described with a Hill function and the reporter enzymatic reaction follows Michaelis-Menten kinetics (see Fig 1B for the reactions presented).

The model equations for the PhyA_FHY1/FHL system are presented below:

(1)

(2)

(3)

(4)

(5)

(6)

(7)

The equations (1)-(5) describe changes in concentrations of *all *the phytochrome components, while (6) and (7) correspond to changes in concentrations of luciferase mRNA and protein, respectively.

Luminescence level is calculated according to the Michaelis-Menten equation:

(8)

Light emission is measured in terms of Relative Light Units (RLU) per second and this corresponds to the rate of the light emission reaction for the colony [28]. The parameter RLU is a conversion factor that translates the number of moles of luciferin reacted into the RLU measurement by the instrument. This also accounts for the discrepancies in colony sizes (Fig 5), growth rate, and instrument characteristics.

### 'Diffusion' part of the model

Our experimental setup involves the application of a relatively small amount of luciferin substrate (20 μl) to a yeast patch, growing in a 100-mm diameter plate on an agar gel of 5-7 mm thickness. We assumed that the initial decrease in luminescence level just after luciferin application predominantly resulted from the diffusion of substrate through the gel. This was confirmed by an additional experiment (Additional file 5). Taking into account that the thickness of the gel is much smaller than the diameter of the plate, we assumed that the diffusion of luciferin could be described with the diffusion equation in polar cylindrical coordinates:



The particular solution of form

(9)

was found to fit experimental data with the best accuracy. Here, S is the cytosolic luciferin concentration, D is the diffusion coefficient, r0 is the effective colony radius, A and B are constants of integration.

#### 2. PHYA_FHL Model Reactions

(1a)

(2a)

(3a)

(4a)

(5a)

(6a)

(7a)

(8a)

(9a)

(10)

(11)

(12)

(13)

(14)

(15)

(16)

(17)

(18)

(19)

### Conservation laws



#### 3. PHYB-PIF3 Model Reactions (via Shimizu-Sato's system)

Model for the Shimizu-Sato's system has the similar structure but differs in reporter -LacZ. Model lacks the description of the reporter protein kinetics due to the stability of LacZ protein and the overall relative shortness of the timescale investigated in the paper (2 h) (See Additional file 4 for the data and model simulation).

(1b)

(2b)

(3b)

(4b)

(5b)

(6b)

(7b)

(8b)

#### 4. Estimation of photoconversion rates

For estimation of photoconversion rates we used the data for the photoconversion cross-section of Pr, Pfr andP and Pfr/P ratios at photoequilibrium of type -I phytochrome [30].

• Ka for R and FR were estimated according to the wavelength in experiment for 660 nm R and 730 nm FR: Ka_R = 4963e-6 (m2/um); Ka_FR = 35.53e-6(m2/um); Ki_R = 743.9e-6(m2/um); Ki_FR = 1701e-6(m2/um)

• Light intensity for the R from experiment conditions is 70 (umol/m2/s) = >70*3600 = 252000(umol/m2/h)

• For FR -is 80 (umol/m2/s) = >80*3600 = 288000 (umol/m2/h)

#### 5. Fitting to experimental results

The model was developed in SBTOOLBOX2 for MATLAB and fitted with a particle-swarm optimisation algorithm from the SBPD package in SBTOOLBOX2 [37,38]. Experiments were designed to cover all possible states of the system that have to be addressed in the model. We started with fitting the model to the simple experimental protocol, including dark conditions and red light application with or without the subsequent immediate far-red application (Fig 6A). Dark experiments taken separately provided us with parameter values for the luciferase system (see Table 1), namely the degradation and translation rates, that were fixed during the following optimization procedure. Light response parameter values were estimated from R and R-FR experiments. For that the model was simultaneously fitted to five sets of ON-OFF experiments, each containing seven experiments: R, dark and five combination of R followed by FR with intervals 0 h, 0.5 h, 1 h, 3 h and 9 h (Fig 6B). Thus, a total of 35 timeseries (each of 210-360 timepoints) were fitted simultaneously. Fitting results demonstrate a good accuracy (see Fig 6A, B) with the root mean square deviation of 1.9*10^-3^.

As we aimed to account for increasing variability arising from solid culture conditions, our model parameters comprise the members which appear specific in each experiment. First of all, this relates to parameters corresponding to 'diffusion' section (D, r0, A and B) as they establish the initial conditions by the time of light treatment. Secondly, parameter RLU that accounts for variability in colony size and growth rate also has to be locally estimated for each experiment. Therefore, besides the parameters identical for all experiments shown in Table 1, the system contains 5 local parameters that have to be fitted in SBPD individually for each timeseries to obtain the results in the same scale.

#### 6. Identifiability analysis

The MOTA tool was used to assess the identifiability of the model and reduce the number of free parameters in the fitting procedure [39]. We started from non-identifiable system with 17 parameters and followed the procedure described in [39]. After we fixed in series the functionally related parameters, we ended up with an identifiable 10-parameter system (Table 1).

• 500 parameter set were obtained from independent fittings from 500 random initial points

• Parameter sets were analysed to find statistical dependence between parameters

• Seven parameters were fixed to make Pool_Phy, k_base, m_luc1, K_rev, g_Luc, K_dis, k_kat

• The Final model contained 10 free parameters

## Competing interests

The authors declare that they have no competing interests.

## Authors' contributions

All authors designed experiments; OS, AK, LED and LKB performed experiments and analysed data; KT constructed materials; OS designed and performed modeling, simulation and analysis; OS, LED, LKB, FN and AJM wrote the paper.

## Supplementary Material

Additional file 1 **Light switch and luciferin pre-treatment**. The data provided demonstrate the behavior of the light switch system from the time point of the luciferin pretreatment. Yeast cells harboring the *GAL1:LUC *reporter and expressing PHYA-GBD/GAD-FHY1 (A), PHYA-GBD/GAD-FHL (B), or PHYBNT-GBD/GAD-PIF3 (C) fusion protein-pairs were grown in darkness to form patches (merged colonies) for two days at 30°C, treated with 2.5 mM luciferin and transferred to 22°C for 17.5 h. Separate yeast patches were irradiated at 17.5 h with single red (R), or far-red (FR) light pulses, or with red pulses immediately followed by far-red pulses (R/FR), or were kept in darkness (Dark). Time zero corresponds to the time of luciferin application +1 h. Absolute luminescence levels corrected for the background of the camera are shown. Click here for file

Additional file 2 **Basal expression level of the *GAL1:LUC *reporter**. The data provided show the basal expression level of the *GAL1:LUC *reporter in yeast patches grown with and without added PCB. Yeast cells harboring the *GAL1:LUC *reporter, but lacking any plasmids expressing PHYA, PHYB, FHY1 or FHL proteins were grown in darkness to form patches for two days at 30°C, treated with 2.5 mM luciferin and transferred to 22°C for 21 h. Separate yeast patches were irradiated at 21 h with single red (R), or far-red (FR) light pulses, or with red pulses immediately followed by far-red pulses (R/FR), or were kept in darkness (Dark). Yeast patches were grown on media with PCB (A) or without PCB (B). Time zero corresponds to the time of luciferin application +1 h. Absolute luminescence levels corrected for the background of the camera are shown. Click here for file

Additional file 3 **Model for PhyA_FHL system in SBML format**. Model in SBML Click here for file

Additional file 4 **Simulation of PhyB-PIF3 system**. The data represent the results of model fitting to the data from Shimizu-Sato et al. [6]. **A**. Far-red reversal timecourse (long-term): Red light (RL) at 0, Far-Red(FRL) at 0.5 h, 1 h, 2 h. **B **Far-red reversal timecourse(short-term): Red light at 0, Far-Red at 0.5 h. Data are presented with dots and model simulation with solid lines. Click here for file

Additional File 5 **Diffusion rate estimation experiment**. The data represent the experiment for the estimation of the diffusion rate. Yeast colonies were grown from 20 μl of OD_600 _0.6 cultures and placed at 1 cm intervals as the pattern indicates in the figure above. Cultures were grown following same conditions as described in Materials and Methods. Luciferin was applied to the central well (orange on figure) and images were taken at 6 min intervals for 26 hours. The rate of diffusion was calculated from the successive time intervals between colonies showing luminescence. Click here for file
