# Supplementary figures and images for: A switchable light-input, light-output system modelled and constructed in yeast
Source: J Biol Eng. 2009 Sep 17;3:15. doi: 10.1186/1754-1611-3-15 (PMC2758823; doi:10.1186/1754-1611-3-15)

## Slide 1
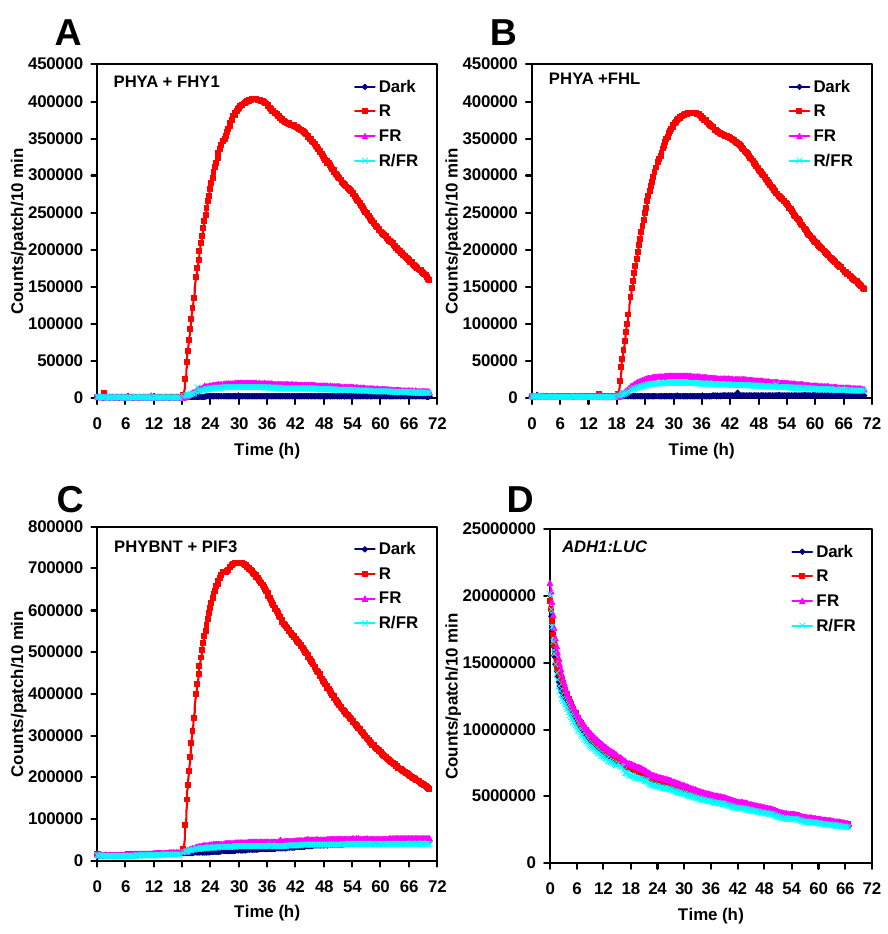

A
B
PHYA +FHL
PHYA + FHY1
C
D
PHYBNT + PIF3
ADH1:LUC

Supplement: Additional file 1 — Light switch and luciferin pre-treatment. The data provided demonstrate the behavior of the light switch system from the time point of the luciferin pretreatment. Yeast cells harboring the GAL1:LUC reporter and expressing PHYA-GBD/GAD-FHY1 (A), PHYA-GBD/GAD-FHL (B), or PHYBNT-GBD/GAD-PIF3 (C) fusion protein-pairs were grown in darkness to form patches (merged colonies) for two days at 30°C, treated with 2.5 mM luciferin and transferred to 22°C for 17.5 h. Separate yeast patches were irradiated at 17.5 h with single red (R), or far-red (FR) light pulses, or with red pulses immediately followed by far-red pulses (R/FR), or were kept in darkness (Dark). Time zero corresponds to the time of luciferin application +1 h. Absolute luminescence levels corrected for the background of the camera are shown. [file 1754-1611-3-15-S1.PPT]

## Slide 1
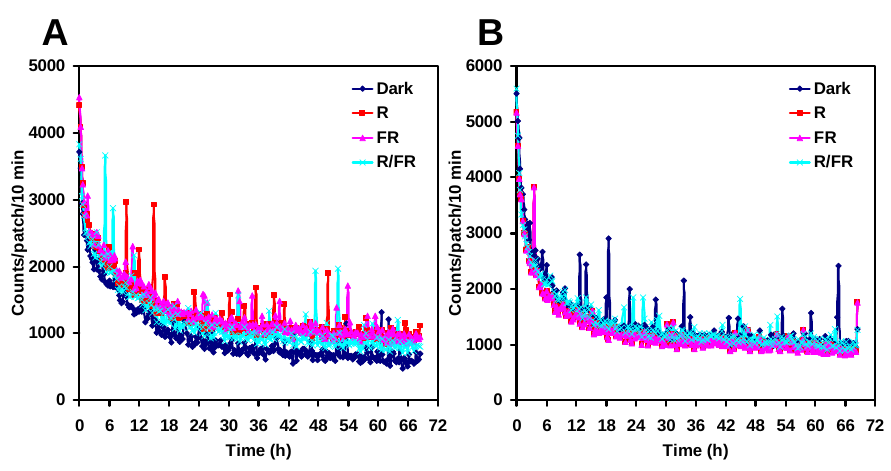

A
B

Supplement: Additional file 2 — Basal expression level of the GAL1:LUC reporter. The data provided show the basal expression level of the GAL1:LUC reporter in yeast patches grown with and without added PCB. Yeast cells harboring the GAL1:LUC reporter, but lacking any plasmids expressing PHYA, PHYB, FHY1 or FHL proteins were grown in darkness to form patches for two days at 30°C, treated with 2.5 mM luciferin and transferred to 22°C for 21 h. Separate yeast patches were irradiated at 21 h with single red (R), or far-red (FR) light pulses, or with red pulses immediately followed by far-red pulses (R/FR), or were kept in darkness (Dark). Yeast patches were grown on media with PCB (A) or without PCB (B). Time zero corresponds to the time of luciferin application +1 h. Absolute luminescence levels corrected for the background of the camera are shown. [file 1754-1611-3-15-S2.PPT]

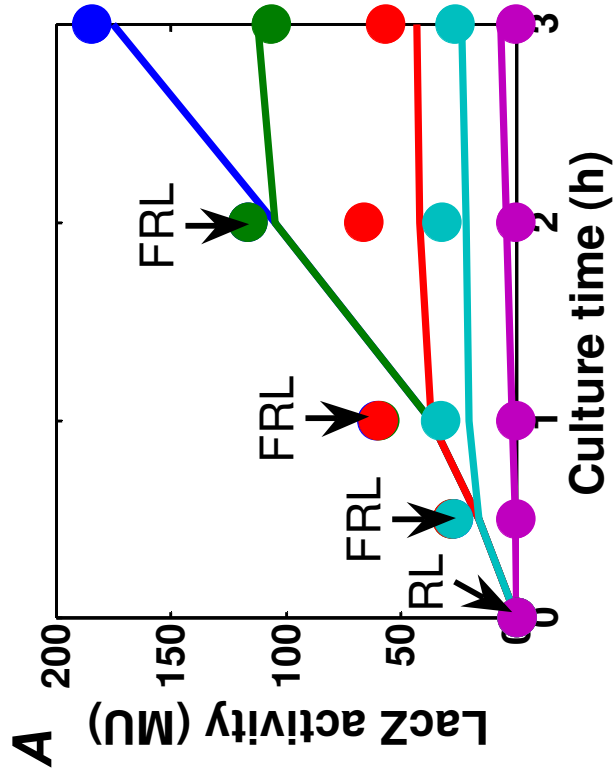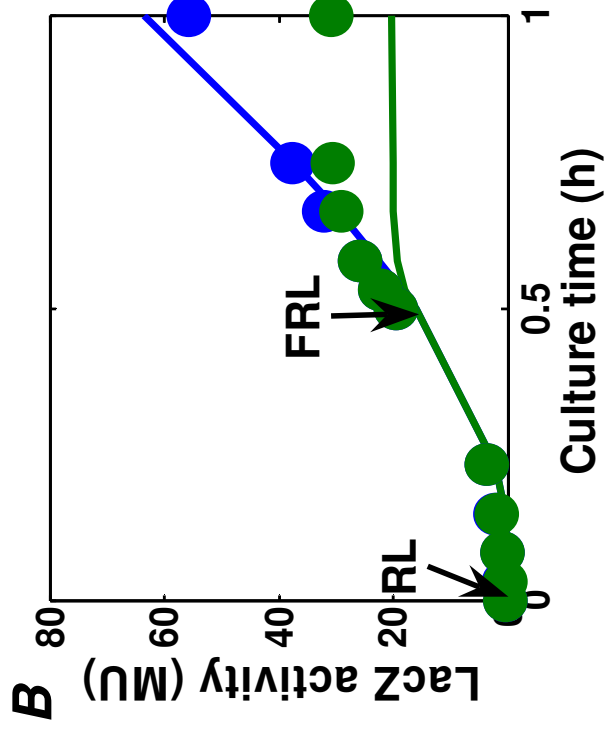

Supplement: Additional file 4 — Simulation of PhyB-PIF3 system. The data represent the results of model fitting to the data from Shimizu-Sato et al. [6]. A. Far-red reversal timecourse (long-term): Red light (RL) at 0, Far-Red(FRL) at 0.5 h, 1 h, 2 h. B Far-red reversal timecourse(short-term): Red light at 0, Far-Red at 0.5 h. Data are presented with dots and model simulation with solid lines. [file 1754-1611-3-15-S4.PDF]

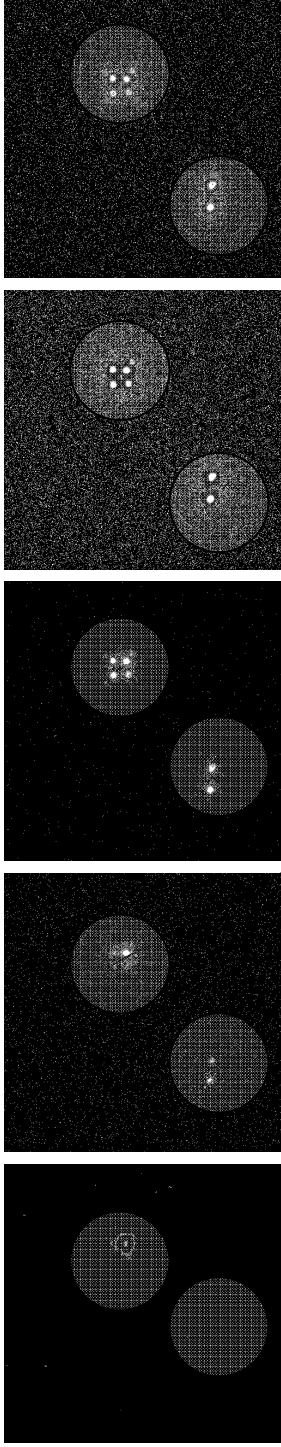

16h

12h

8h

4h

0h

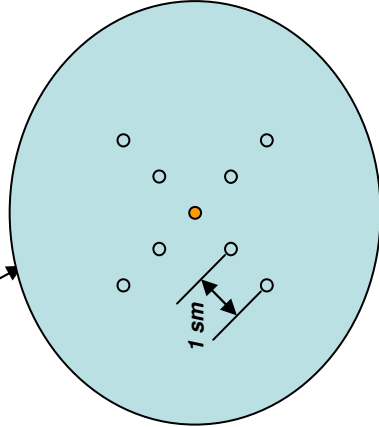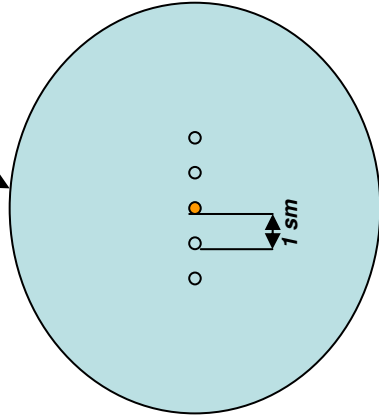

Supplement: Additional File 5 — Diffusion rate estimation experiment. The data represent the experiment for the estimation of the diffusion rate. Yeast colonies were grown from 20 μl of OD600 0.6 cultures and placed at 1 cm intervals as the pattern indicates in the figure above. Cultures were grown following same conditions as described in Materials and Methods. Luciferin was applied to the central well (orange on figure) and images were taken at 6 min intervals for 26 hours. The rate of diffusion was calculated from the successive time intervals between colonies showing luminescence. [file 1754-1611-3-15-S5.PDF]
